# The G_1 _phase Cdks regulate the centrosome cycle and mediate oncogene-dependent centrosome amplification

**DOI:** 10.1186/1747-1028-6-2

**Published:** 2011-01-27

**Authors:** Mary K Harrison, Arsene M Adon, Harold I Saavedra

**Affiliations:** 1 Emory University, Department of Radiation Oncology, Winship Cancer Institute, 1701 Uppergate Drive, Atlanta, Georgia, 30322, USA

## Abstract

Because centrosome amplification generates aneuploidy and since centrosome amplification is ubiquitous in human tumors, a strong case is made for centrosome amplification being a major force in tumor biogenesis. Various evidence showing that oncogenes and altered tumor suppressors lead to centrosome amplification and aneuploidy suggests that oncogenes and altered tumor suppressors are a major source of genomic instability in tumors, and that they generate those abnormal processes to initiate and sustain tumorigenesis. We discuss how altered tumor suppressors and oncogenes utilize the cell cycle regulatory machinery to signal centrosome amplification and aneuploidy.

## The centrosome and cancer

It has well been established that centrosome amplification is a distinct feature of most cancer cells. With this observation came the hypothesis that this phenotype can drive genomic instability and subsequent tumorigenesis. Abnormal centrosome biology, including centrosome amplification and structural abnormalities frequently occurs in most types of solid tumors, as well some leukemias and lymphomas. Specifically, those cancer types include testicular germ cell, liposarcoma, adrenocortical, bronchial, bladder, cerebral primitive neuroectodermal, cervical, prostate, breast, squamous cell carcinomas of the head and neck, myeloma, and T-cell leukemia [1-13]. Work done in haematopoietic malignancies demonstrates that centrosome amplification in myelomas correlates with a specific gene expression signature, and can serve as a prognostic factor in patients [14].

One of the tumor types in which the relationship between centrosome amplification and cancer is better understood are breast cancers. The vast majority (80-100%) of breast tumors display centrosome amplification [15]. Breast adenocarcinoma cells have a much higher frequency of centrosome defects, including amplification of number [15,16], increased volume and supernumerary centrioles, when compared to normal breast tissue [16]. Similar phenotypes can also be found in pre-invasive *in situ *ductal carcinoma, and in pre-malignant breast lesions, suggesting that these aberrations occur early in breast carcinogenesis [4,15,17]. In support of this data, molecular analyses have found that the centrosome pathway is highly enriched for SNPs that are associated with breast cancer risk [18]. In addition to being involved in initiation, having extensive areas of centrosome amplification in breast tumors correlates with axillary lymph node involvement, suggesting that centrosome amplification also contributes to the most malignant characteristics of breast cancer cells [19]. Various rodent models have also given support to the idea that centrosome amplification is involved in mammary tumor initiation. For example, treatment of female Wistar-Furth rats with MNU leads to mammary tumorigenesis. MNU-induced preneoplastic lesions exhibited DNA damage, chromosomal instability, and supernumerary centrosomes [20]. Additionally, expression of Pin1 in the mammary epithelial cells of transgenic mice leads to hyperplastic lesions harboring centrosome amplification [21]. Also, our laboratory has recently shown that inducible expression of K-Ras^G12D ^results in mammary hyperplasias that harbor centrosome amplification, thus demonstrating that centrosome amplification precedes mammary tumorigenesis [22].

Therefore, there are many similar correlative studies that link centrosomal abnormalities and cancer, and there are even more studies working to discover the causal link and mechanism behind this well established correlation. Indeed, the most direct evidence showing that centrosome amplification is involved in tumorigenesis was obtained in *Drosophila*. In a study that specifically addressed the relationship between abnormal centrosome biology and tumorigenesis, Basto et al. assayed the long term consequences of an organism having supernumerary centrosomes. Allotransplantation of Plk4/SAK over-expressing *Drosophila *neuronal stem cells is sufficient to induce tumors in flies [23]. Also, transplanted cells expressing *aur-a, plk, asl *and *dsas4 *resulted in tumors with varying efficiency [24]. Aurora A, one of the first oncogenes shown to induce centrosome amplification in mammalian cells [25], proved to be the most efficient at inducing tumors [24]. These important experiments and observations are the first step in defining the link between centrosome amplification and tumors. This review will address how the G1 phase Cdks normally regulate the centrosome cycle, and how oncogenes and tumor supressors deregulate those Cdks to signal centrosome amplification.

## The coordinated activities of G_1 _phase Cdks, centrosomal kinases and phosphatases regulate the centrosome cycle

### The centrosome duplication cycle

It can be argued that faithful segregation of chromosomes into daughter cells during mitosis is essential to maintain genetic stability in most if not all organisms. The interplay between centrosomes and the mitotic microtubules results in the accurate segregation of chromosomes into daughter cells. Following cytokinesis each daughter cell receives only one centrosome; this centrosome, like DNA, must duplicate only once prior to the next mitosis. Centrosome duplication must be tightly regulated, because the generation of more than one procentriole per mother centriole results in centrosome amplification [26,27] and contributes to tumorigenesis [23,24]. The different phases of the centrosome cycle were originally assigned based on the morphology of the centriole pair throughout the cell cycle, as established by electron microscopy [28]. More recently, establishment of centriole duplication assays in *Xenopus *egg extracts [29] and cultured mammalian cells [30,31] remarkably improved the dissection of the centrosome cycle. Additionally, the development of centrin-2-GFP constructs has allowed following the centrosome duplication cycle relative to the different cell cycle phases in real-time [32], and allows the assessment of unregulated centrosome cycles [33].

Laser centrosomal ablation and mutants of *Chlamydomonas *that are defective in centriole segregation showed two pathways for centriole assembly, namely a template pathway that requires preexisting centrioles to nucleate new centriole assembly, and a *de novo *assembly pathway that is normally turned off when centrioles are present [34,35]. The templated pathway occurs as follows [36,37]: Throughout early G_1 _phase, normal cells have one mature centrosome. During late G_1 _and S phase, the structure of the mother and daughter centrioles differs, the mother centriole contains appendages, whereas the daughter centriole grows throughout these phases. At the beginning of S phase, centriole duplication starts with the appearance of short daughter centrioles, or procentrioles, at right angles to the two original centrioles [36,38]. Procentrioles are observed approximately 4 hours after the beginning of S phase [39]. This process culminates in the acquisition of appendages by the daughter centriole in G_2 _[37] and the recruitment of PCM [36,38]. By late G_2_, two mature centrosomes are generated. The *de novo *assembly pathway is first detected by the appearance of small centrin aggregates at S phase [40]. Formation of new centrosomes subsequently occurs in two steps. First, approximately 5-8 hours after centrosome ablation, clouds of pericentriolar material (PCM) containing γ-tubulin and pericentrin appear in the cell [41]. By 24 hours centrioles have formed inside of the already well-developed PCM clouds.

Recent studies identifying several centrosome-associated proteins, protein kinases and phosphatases have provided new insights into the regulation of centrosome structure and function, including their ability to control centriole duplication. Because unregulated expression of proteins controlling the synthesis of daughter centrioles can cause centriole reduplication and centrosome amplification, these proteins are potential targets of oncogenes and altered tumor suppressors, and will be thoroughly discussed in the following sections.

### The G_1 _phase Cdks coordinate the cell and centrosome cycles

The centrosome duplication cycle must occur in coordination with the cell cycle; otherwise, unregulated centrosome duplication may culminate in centrosome amplification. Because DNA and centrosomes undergo semi-conservative duplication once every cell cycle, mammalian cells are equipped with a mechanism that coordinates these two events, so that they are duplicated only once [26]. This coordination is in part accomplished because cell cycle regulatory proteins also regulate the centrosome duplication cycle. The cell cycle is regulated as follows: The temporal overexpression of cyclins D, E, and A sequentially activates the G_1 _phase Cdks, Cdk4/Cdk6 and Cdk2, to trigger entry and progression through S phase [42-51]. The G_1 _phase Cdks trigger the initiation of DNA duplication in part through the phosphorylation of the retinoblastoma (Rb) protein and the activation of the E2F transcriptional program [49,52-73]. The Rb/E2F transcription program is essential for the correct expression and regulation of copious genes involved in DNA replication, DNA repair, mitosis and centrosome duplication [74-76].

Other studies have shown a close relationship between cell cycle regulatory molecules and the regulation of centrosome duplication. For example, ectopic expression of the cyclin-dependent kinase inhibitors p21^Waf1/Cip1 ^and p27^Kip1 ^blocked centrosome duplication in *Xenopus *dividing embryos at the blastomere stage [77]. In support of those studies, inhibition of cyclin E/Cdk2 in *Xenopus *egg extracts caused arrest in S phase and thus prevented centriole re-duplication; re-introduction of cyclin E/Cdk2 restored that reduplication [29]. It was then suggested, using the same system, that inhibition of Cdk2 activity prevents multiple rounds of centriole duplication, but it does not prevent the initial round of duplication [78]. However, there is other more recent evidence suggesting that Cdk2 is also involved in the initial round of centriole duplication. In *Xenopus *egg extracts, separase causes disengagement of centrioles during anaphase, and cyclin E/Cdk2 activity is required for the synthesis of a daughter centriole following disengagement [79].

Although various data obtained in *Xenopus *provided a strong correlation between Cdk2 activity and centrosome duplication, gene knockout experiments done in mammalian cells uncovered a much different scenario. Previous studies demonstrating that Cdk2-deficient mice develop rather normally [80,81], raised the question of the requirement of Cdk2 in other processes such as its ability to regulate DNA and centrosome duplication [80-82]. A surprising result was that cells derived from these mice can proliferate and undergo centrosome duplication with moderate defects [80-82], indicating that the function of Cdk2 for proliferation and initiation of the centrosome duplication can be readily and functionally replaced by other Cdks or other centrosome regulatory proteins. Likewise, ablation of the Cdk2 activating partners cyclin E1 and E2 in mouse embryonic fibroblasts was not associated with any centrosomal defects [83]. In support of studies done in mammalian cells, various combinatorial knockdowns of two mitotic cyclins (CycA, CycB, and CycB3), and reduction of the dosage of the remaining cyclins in *Drosophila *embryonic syncytial divisions allows centrosomes to duplicate, while cells do not enter mitosis [84].

Recent experiments have revealed both redundancy, as well as specificity, in regards to the G_1 _phase Cdks regulating centrosome duplication in eukaryotes. For example, chicken DT40 mutants were generated in which an analog-sensitive mutant *cdk1 *replaced the endogenous *Cdk1*. In those cells, Cdk1 could be inactivated using bulky ATP analogs [85]. In DT40 cells that also lack Cdk2, Cdk1 activity is essential for DNA replication initiation and for centrosome duplication. Also, the relative contributions of the G_1_-Cdks (Cdk2 and Cdk4) to regulate normal centrosome duplication were explored [86]. During these studies, experiments used to measure the centrosome cycle at various time points throughout the cell cycle in *Cdk2*^-/- ^and *Cdk4*^-/- ^MEFs, as well as transient down-regulation of Cdk2 and Cdk4 using RNA-mediated interference, uncovered distinct centrosome cycle defects, suggesting that Cdk2 and Cdk4 do not have redundant functions. For example, while *Cdk2 *deficiency allowed the separation and duplication of centrosomes, absence of *Cdk4 *favored the accumulation of cells with centrosomes that were slow to separate and duplicate.

### Targets of the G_1 _phase Cdks

**Figure 1:**
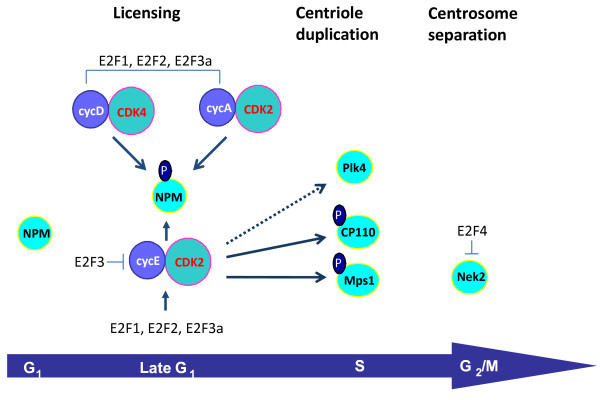
**The G_1 _phase Cdks and the E2Fs regulate various steps in the centrosome duplication cycle**. Various evidence suggests that the G_1 _phase Cdks directly phosphorylate NPM, CP110 and Mps1 to regulate centrosome licensing and duplication. The dotted line reflects the fact that even though Plk4 is not a direct target of Cdk2, introduction of a dominant-negative Cdk2 construct renders it ineffective in triggering centriole reduplication. The figure reflects how the E2F activators E2F1, E2F2 and E2F3 influence the centrosome duplication cycle by controlling the transcriptional levels of cyclins E, A, D, and Cdk2. The figure also reflects how E2F3 and E2F4 repress cyclin E and Nek2 to influence the centrosome cycle.

There are many structural proteins, kinases and phosphatases that regulate centrosome duplication both dependent on and independently of the G_1 _phase Cdk/Rb pathway [87,88]. However, those regulatory molecules acting independently of the G_1 _Cdks will not be covered in the scope of this review. One mode of regulation of centrosome duplication carried out by the G_1 _phase cyclins/Cdks is the phosphorylation of Rb family members, thus triggering de-repression and activation of E2F-responsive genes [33,74-76]. E2F-dependent centrosome regulatory targets target genes including cyclin D1 [89], cyclin E [74,90], cyclin A [76,91], Cdk2 [74], Nek2 [76], and RanBPM [76]. However, this mode of regulation remains poorly understood. A summary of known E2F targets that are known to be involved in the regulation of the centrosome cycle is presented in Figure 1.

A mode of regulation that is more clearly understood is the ability of the G_1 _phase Cdks to phosphorylate centrosome regulatory targets modulating centrosome duplication. For example, nucleophosmin (NPM), also known as B23 [92], numatrin [93], or NO38 [94], was originally identified as a nucleolar phosphoprotein found at high levels in the granular regions of the nucleolus. NPM is a negative suppressor of licensing the centrosome cycle, and a suppressor of centrosome amplification. This was demonstrated using a genetic approach; haploinsufficiency of NPM results in unregulated centrosome duplication and centrosome amplification [95]. Conversely, microinjecting an antibody against NPM results in the suppression of centrosome duplication [96]. Licensing is modulated by G_1 _phase Cdks through phosphorylation and inactivation of NPM, as expression of NPM/B23 mutants whose phosphorylation sites were either deleted (NPM∆186-239) or replaced with a non-phosphorylatable residue (NPM T199A) resulted in suppression of centrosome duplication. NPM is a primary target of Cdk2/cyclin E during the initiation of centrosome duplication (Figure 1) [96]. Cdk2/cyclin A is also known to phosphorylate NPM/B23 specifically on Thr199 *in vitro *at a similar efficiency with Cdk2/cyclin E [97]. In addition, Cdk4/cyclinD also phosphorylates NPM on Thr 199 at mid/late G_1 _phase of the cell cycle [86]. NPM associates specifically with unduplicated centrosomes and dissociates from centrosomes upon Thr199 phosphorylation by Cdk2/cyclin E at the late G_1 _phase [96]. It is believed that the continual presence of active Cdk2/cyclin A may be responsible for preventing re-association of any cytoplasmic NPM/B23 to centrosomes during S and G_2 _phases. During mitosis, NPM/B23 re-associates with the centrosomes and the spindle poles [96,98]; the phosphorylation of NPM/B23 by Cdk1/cyclin B on Thr 234 and/or Thr 237 sites may play a role in re-association of NPM/B23 with centrosomes during mitosis [97]. More recently, it has been shown that NPM is also downstream of other signaling pathways, as phosphorylation of NPM by Plk2 is critical to centrosome duplication [99]. Also, NPM prevents centrosome amplification by forming a complex with BRCA2 and ROCK2 [100].

Some of the first evidence showing that centrosomal kinases are responsible for various steps in the centrosome duplication cycle was obtained from studies on the spindle pole body (SPB), the centrosome-like organelle in yeast. Like the centrosome in other organisms, the SPB duplicates only once per cell cycle commencing in G_1_, an event necessary for the formation of a normal bipolar spindle [101]. The Mps1 (mono polar spindle 1) family was first described in budding yeast based on its mutant phenotype, the formation of a monopolar spindle as a consequence of the failure to duplicate the SPB [102]. Localized to SPBs, Mps1 acts to control their assembly [103]. In mammalian cells, a homologous protein Mps-1 is also involved in centriole duplication. Normally, NIH3T3 cells arrested in S phase undergo only a single round of centrosome duplication [104]. In contrast, overexpression of mMps1p in these cells induced centrosome reduplication, and transfection of mMps1-KD (kinase dead) in these and other cell types (CHO, U20S) blocked centrosome duplication. The turnover of Mps1 kinases through protein degradation may be an important mechanism for their control. For example, stabilization of mMps1p within centrosomes is thought to be achieved by direct phosphorylation of mMps1p by Cdk2 (Figure 1) [104], as overexpression of cyclin A or brief proteasome inhibition increases the centrosomal levels of Mps1, whereas depletion of Cdk2 leads to the proteasome-dependent loss of Mps1 from centrosomes [105]. Also, when a Cdk2 phosphorylation site within Mps1 (T468) is mutated to alanine, Mps1 cannot accumulate at centrosomes or participate in centrosome duplication. In contrast, phosphomimetic mutations at T468 or deletion of the region surrounding T468 prevent the proteasome-dependent removal of Mps1 from centrosomes in the absence of Cdk2 activity. Moreover, cyclin A-dependent centrosome reduplication requires Mps1. Although Mps1 was reported to be involved in centrosome duplication with Cdk2 as the downstream regulator [104], another report concluded that human Mps1 does not localize to centrosomes and is not required for the ability of human U2OS cells to undergo centrosome reduplication [106]. Interestingly, it was recently shown that human Mps1 (hMps1) localizes to centrosomes after the staining of a variety of human cell types with an antibody specific to hMps1 [107]. These studies also demonstrated that overexpression of kinase dead hMps1 blocked centrosome duplication in NIH3T3, HeLa, RPE1and U2OS, and that transfection of hMps1 in U2OS cells accelerated centrosome reduplication. They also showed that siRNA silencing of hMps1 in HeLa cells induced failures in both centrosome duplication and normal progression of mitosis.

Cdk2 is responsible for regulating other proteins involved in centrosome duplication, although it is still not clear how Cdk2 controls their activity. For example, in mammalian cells, Plk4 cooperates with Cdk2, CP110 and Hs-SAS6 to induce centriole duplication [108]. Although Plk4 has not been reported to be a direct Cdk2 phosphorylation substrate, Plk4's centriole duplication activity is inefficient in the presence of a Cdk2 dominant-negative construct (Figure 1). Also, a screen for various substrates of Cdk2 revealed that CP110 is a target of Cyclin E/Cdk2, Cyclin A/Cdk2 and of Cyclin B/Cdc2 (Figure 1) [109]. CP110 is regulated by the cell cycle, as it is induced at G_1_/S phase, and its mRNA levels are suppressed after S phase. Down-regulation of CP110 with siRNA suppressed centriole reduplication in HU-treated U2OS cells; also, cells expressing CP110 lacking Cdk phosphorylation sites, or down-modulated CP110 also displayed centrosome separation. However, even though these studies revealed that CP110 is involved in centriole duplication and centrosome separation, the individual contribution of Cdk2 and Cdc2 sites in regulating those processes remains to be addressed.

## Deregulated G_1 _Cdks, centrosome amplification and cancer

### Oncogene-dependent centrosome amplification correlates with hyperactive Cdk2 and Cdk4

Because the centrosome cycle is regulated in part by cell cycle machinery, when the cell cycle becomes deregulated by oncogenes and altered tumor suppressors, the centrosome can also be susceptible to deregulation. This can ultimately lead to centrosome amplification, aneuploidy, and unregulated cell cycling [110,111]. Mounting evidence is showing that uncontrolled G_1 _phase cyclin/Cdk complexes affect two major steps in the centrosome cycle: licensing and centriole duplication.

Alterations to the centrosome duplication machinery can lead to centriole reduplication, defined as the generation of multiple procentrioles from one mother centriole; this often results in centrosome amplification. Deregulated centriole duplication and centrosome amplification was addressed using laser microsurgery to show that physical removal of all over-duplicated daughter centrioles induces reduplication of the mother in S-phase-arrested cells CHO cells [112]. In a subset of mammalian cells lacking checkpoint controls, including Chinese hamster ovary (CHO) cells [30], or *p53*^-/- ^mouse embryonic fibroblasts [86], hydroxyurea (HU) treatment arrests the cells in S phase while centrosome duplication continues and results in centriole reduplication. In contrast, in CHO cells treated with mimosine, both the cell and centrosome cycles are arrested. Using that system, experiments showed that Cdk2 activity was higher in HU-treated cells than in mimosine-treated cells, suggesting a strong correlation between increased Cdk2 activity and excessive centriole duplication [30]. Also, more recent studies have shown that CHO cells arrested in G_1 _with mimosine can also assemble more than four centrioles, but the extent of centrosome amplification is decreased compared to cells that enter S-phase and activate the Cdk2-cyclin complex [113]. In mammalian somatic cells, centrosome reduplication is attributed to the Cdk2/cyclin A complex, since overexpression of cyclin A in cells arrested in S phase (by the expression of p16, non-phosphorylatable Rb, or in cells treated with HU), triggers centriole reduplication, while a Cdk2 dominant negative blocks reduplication [31]. Also, ectopic expression of E2F2 or E2F3 can relieve that block, suggesting that centriole re-duplication is in part mediated downstream of Cdk2 and Rb.

The first altered tumor suppressor shown to be directly associated with centrosome amplification was p53, as its genetic deletion in mouse embryonic fibroblasts promoted that abnormal process [114]. Similarly, alterations that affected p53 function resulted in centrosome amplification. For example, MDM2, an E3 ubiquitin ligase that promotes degradation of p53 [115], associates with centrosome amplification in squamous cell carcinomas of the head and neck (SCCHN) [5]. Also, the E6 viral protein from the HPV16 virus, which inactivates p53, causes centrosome amplification [116]. One of the most important functions of the p53 pathway is to trigger cell cycle arrest to allow repair of DNA damage, or cell death if the damage is unrepaired [117]. p53 exerts some of its cell cycle regulatory functions through promoting the transcription of p21^Waf1/CIP1^, a CKI that negatively regulates both Cdk2 and Cdk4 activities [118,119]. p53 prevents centrosome amplification through direct binding to the centrosome, and also in part through its ability to regulate p21^Waf1/CIP1 ^[120]. Several groups have presented data supporting a role of p21^Waf1/CIP1 ^in centrosome biology. For example, introduction of p21^Waf1/CIP1 ^into p53^-/- ^cells harboring centrosome amplification restored normal centrosome duplication and abrogated centrosome amplification [121]. Moreover, knock-down of p21^Waf1/CIP1^in murine myeloblasts stimulates excessive centriole numbers in the presence of only one mature centriole [122] and p21^Waf1/CIP1 ^null human hematopoietic cells display elevated frequencies of centrosome amplification [123].

Consequent to the discovery that centrosome amplification in p53-null cells correlated with deregulated Cdk2 activity, many other studies began showing similar correlations. For example, when E2F3a/b, transcription factors critical to S phase entry, are ablated, elevated cyclin E-dependent Cdk2 activity correlates with constitutive centriole separation, duplication, and centrosome amplification (Figure 1) [33]. It is to note that this function is specific to E2F3-null cells, as MEFs lacking E2F1, E2F2, E2F4 or E2F5 do not display centrosome amplification. Also, the expression of the centrosome-targeting region of CG-NAP (a centrosome and Golgi-localized protein), causes centrosome amplification by anchoring excess amount of cyclin E-cdk2 to centrosomes [124]. In another correlative study disruption of Skp2, a substrate recognition component of an Skp1-Cullin-F-box protein (SCF) ubiquitin ligase, results in increased cyclin E, p27, and centrosome amplification [125]. Another example is ECRG2, a novel tumor suppressor gene which localizes to centrosomes; its depletion destabilizes p53, leading to down-regulated p21, increased cyclin E/Cdk2 activity, and centrosome amplification [126]. On the other hand, there are proteins that prevent excessive centriole duplication triggered by de-regulated G_1 _phase cyclins. For example, the Orc1 protein, a subunit of the origin recognition complex (ORC) that is a key component of the DNA replication licensing machinery, controls centriole and centrosome copy number in human cells [127]. Cyclin A promotes Orc1 localization to centrosomes, where Orc1 prevents Cyclin E-dependent reduplication of both centrioles and centrosomes.

Following the discovery that tumor suppressors maintained normal centrosome numbers, various laboratories showed that certain protooncogenes displayed the same activity. Some of the first observations that protooncogenes, including tyrosine kinase receptors, controlled the centrosome cycle were made in CHO cells cultured in the presence of hydroxyurea (HU) or aphidicolin. Addition of dialyzed serum to these cells stopped centriole reduplication, while addition of EGF re-initiated the process [128]. Additionally, when PTEN^-/- ^neural precursor cells were infected with retrovirus encoding constitutively active EGFRvIII, centrosome amplification, genomic instability and glial tumors developed [129]. Furthermore, it has been shown that other EGFR family members may play a role in this story. Her2/*neu *(ErbB2) was first described as an oncogene when isolated from neuroglioblastomas that developed in rats treated with ethylnitrosourea (ENU) [130]. Her2 mutations are relatively rare in human cancers; however wild type ErbB2 is amplified at the genomic level or overexpressed at the protein level [131] in approximately 30% of invasive ductal breast cancers [132]. It has been shown that overexpression of this protein correlates with tumor size, spread to lymph nodes, high grade, increased percentage of S phase cells, and aneuploidy [132]. A study of mice expressing activated Her2/*neu *in the mammary epithelium demonstrated its ability to induce chromosomal aberrations as well as centrosome amplification in cell lines derived from primary tumors [133]. Also, analysis of fine-needle aspirations of the breast found a significant correlation between the percentage of cells with centrosome amplification, over-expression of HER2/*neu *and negative ER status [15]. The molecules downstream of Her2 can also become deregulated upon over-expression. Her2 induces cyclin D1 through the Ras/Rac/Rho pathway in which the ERK, JNK and p38MAPK cascades are distal mediators.

Another oncogene that has been associated with centrosome amplification is Ras. A Pubmed search for "Ras and Cancer" returns almost twenty thousand hits for articles and reviews, most discussing the oncogenic potential of Ras and the many cellular phenotypes that it affects. Probably one of the most thoroughly studied of the many Ras-mediated pathways is the MAP kinase cascade, a critical signaling cascade regulating cell proliferation by exerting control over the cell cycle. It has been shown that constitutive activation of MAPK induces defects in the normal mitotic processes of the cell [134]. For example, transduction of v-*ras *or v-*mos *into NIH 3T3 cells induced centrosome amplification and inhibition of this phenotype was possible with the introduction of MAPK inhibitors [134]. A study focusing on genomic instability in thyroid PCCL3 cells harboring wt p53, examined the effects of H-RAS^V12 ^and activated MEK1 and found that both induced centrosome amplification and chromosome misalignment [135]. Likewise, expression of the H-Ras^G12V ^or the H-Ras^G12V ^& c-Myc oncogenes in non-transformed MCF10A human mammary epithelial cells results in elevated frequencies of centrosome amplification [22]. Activation of this pathway is relevant *in vivo*, as ectopic expression of the K-Ras^G12D ^oncogene in mouse mammary epithelial cells resulted in centrosome amplification that greatly preceded tumorigenesis [22].

The extracellular regulated kinase (ERK) cascade, a major component of the MAPK pathway, is a critical signaling cascade, regulating cell proliferation by exerting control over the cell cycle. MEK1 and MEK2, two kinases upstream of ERK, have been shown to regulate cell cycle progression in two distinct ways [136]. Loss of MEK2 results in a mitotic delay, perhaps due to a reduction in ERK phosphorylation. When MEK2 is knocked down using siRNA in HCT116 colon cancer cells, cyclin D1 levels increase, leading to hyperactive Cdk4/6 and hyperphosphorylation of nucleophosmin (NPM); this hyperphosphorylation was independent of Cdk2. Hyperphosphorylation of NPM at T199 was accompanied by centrosome amplification and the appearance of multipolar spindles [136], making a case for Cdk4 mediation of NPM phosphorylation. In another study associating Ras/MAPK to centrosome amplification, the Hepatitis B virus (HBv) was shown to activate various signaling pathways, one of which is the Ras-Raf-MAPK [137]. The hepatitis B virus X oncoprotein HBx, is a small oncoprotein that is required for viral replication and has been associated with HBV-mediated hepatocellular carcinoma. Yun et al. discovered that the Ras-MAPK pathway is the downstream effector of HBx protein involved in abnormal amplification of centrosomes [137]. Suppression of the ERK pathway with inhibitors, and the introduction of dominant negative mutants of Ras and Mek reduce the frequency of supernumerary centrosomes in HBx expressing human Chang liver cells, thus further clarifying the role of Ras and the MAPK pathway in the HBx mediated induction of centrosome amplification [137].

Transcription of the *cyclin D1 *gene and subsequent interaction with its kinetically active partner, Cdk4, depends on receptor mediated Ras signaling. Various upstream and downstream effectors of the MAPK pathway up-regulate the transcription of *cyclin D1 *so that when it is bound to Cdk4 it is able to sequester p27^Kip1 ^and thus activate cyclin E-Cdk2 complex [138]. Upon this activation, both cyclin-Cdk complexes are free to phosphorylate RB family proteins and cells may progress from G_1 _to S phase of the cell cycle [138]. In normal cells mitogenic growth factors are responsible for inducing cyclin D1; however, over-expression of cyclin D1, independent of growth factor signaling, is a common feature of many tumors [138]. For example, a great majority of small cell lung cancers, breast cancers, glioblastomas and mantle cell lymphomas have over-expression of cyclin D1 or its catalytic partner, Cdk4. In fact aberrant over-expression of cyclin D1 occurs in 70-100% of breast tumor cell lines and most breast cancers and seems to be required for *neu *and Ras-induced mammary epithelial transformation [89]. Along the same line, cyclin D and Cdk4 are required for *neu *and *ras *induced mammary tumorigenesis [139,140], demonstrating that the cyclin D1/Cdk4 complex is needed for mammary transformation. Unregulated expression of cyclin D1 is associated with chromosomal abnormalities and it has been documented that transient expression of cyclin D1 in hepatocytes and human mammary epithelial cells induces centrosome amplification [141]. A striking feature of this study demonstrated that centrosome abnormalities persist in a small percentage of the cells for four months after cyclin D1 is no longer expressed [141]. Interestingly, hepatocytes from Cdk2^-/- ^mice are refractive to cyclin D1-dependent centrosome amplification, suggesting that in some contexts, either cyclin D1 uses Cdk2 to trigger centrosome amplification, or that Cdk2 is a downstream target of cyclin D/Cdk4 [142].

In support of the studies linking cyclin D1/Cdk4 with centrosome amplification, one of the primary events associated with initiation of mammary tumorigenesis is the loss of the Cdk4/Cdk6-specific inhibitor p16^INK4A ^through hypermethylation of its promoter, which de-regulates the centrosome cycle and lead to a moderate increase in frequencies of centrosome amplification [143-145]. Concomitantly, the γ-tubulin gene is amplified [146]. Likewise, silencing the histone H3 lysine 9 methyltransferase G9a leads to centrosome amplification, reportedly by down-modulation of gene expression, including that of p16^INK4A ^[147]. Thus, it has been postulated that loss of p16 expression coupled with increased γ-tubulin contributes to centrosome amplification and breast cancer progression.

### Direct evidence demonstrating involvement of the G_1 _phase Cdks in centrosome amplification

Although the evidence associating hyperactive G_1 _phase cyclin/Cdks and centrosome amplification is convincing, it is nevertheless correlative. This is due to the fact that some of the protooncogenes, tumor suppressors, and transcription factors that control G_1 _phase Cdk activities, such as Her2, Ras, E2f3 and p53, also regulate a plethora of other gene products [74,76,148,149]. Table 1 lists a subset of oncogenes and altered tumor suppressors, and the G1 phase Cdk they may hyperactiate to signal centrosome amplification. How do G_1 _phase-CDKs signal oncogene-dependent centrosome amplification? Research showing that inhibition of specific Cdks blocks centriole reduplication was the first direct evidence of a relationship between Cdks and centrosome amplification. In HU-arrested cells, cells treated with butyrolactone I or roscovitine -inhibitors of Cdk2, Cdc2 and Cdk5 activity- [150,151], and cells treated with the Cdk2/Cdk4 inhibitor p21^Waf1/Cip1 ^centriole reduplication was blocked [30]. Following these initial experiments, combinatorial cyclin E/A/p53 gene knockout analyses demonstrated that the G_1 _phase cyclins and Cdks play pivotal roles in signaling centrosome amplification. For example, in p53^-/- ^cells arrested in early S phase, cyclin E, but not cyclin A, is important in centriole reduplication and centrosome amplification, but in the absence of cyclin E, cyclin A can drive the abnormal phenotype [152]. In p53^-/- ^cells, Cdk2 mediated HU-induced centriole reduplication [153]. In another study, centriole reduplication triggered by the peptide vinyl sulfone proteasome inhibitor Z-L(3)VS is dependent on cyclin E/Cdk2, as well as Polo-like kinase 4 [154]. Furthermore, inhibitors of Cdk2, dominant negative mutants of Cdk2 and DP1, siRNA-mediated silencing of Cdk2, or genetic deletion of Cdk2 abrogate centrosome amplification triggered by ectopic expression of E7 [82]. These studies provided direct support to the role played by E2Fs and Cdk2 in centrosome amplification associated with the inactivation of Rb by its conditional loss [155], the acute loss of pRb by adenovirus carrying shRNA against Rb [156], or through the expression of the E7 viral protein from the HPV16 virus [116].

**Table 1:** Oncogenes and inactive tumor suppressors and the G_1 _phase Cdk they may deregulate to signal centrosome amplification.

Genetic alteration	Deregulated Cdk	Reference
**Oncogenes**		
Cyclin D1	Cdk2, Cdk4	[141,142]
ErbB2	Cdk4	[139]
Ras	Cdk4	[22,140]
		
**Tumor Suppressors**		
E2F3a/b	Cdk2	[33]
MEK2	Cdk4, Cdk6	[136]
p16^INK4A^	Cdk4, Cdk6	[143,145]
p21^Waf1/CIP1^	Cdk2, Cdk4	[118,119,121,122]
p53	Cdk2, Cdk4	[86,120,121]
Skp2	Cdk2	[125]
Rb	Cdk2	[82]

Even though most evidence demonstrated that Cdk2 was the central mediator of oncogene-induced centrosome amplification, our group demonstrated that Cdk4 is also an important mediator. For example, genetic ablation of Cdk2 and Cdk4 abrogated centrosome amplification in p53-null cells [86] by restricting NPM-dependent excessive licensing of the centrosome cycle, as well as by restricting centriole reduplication in *p53*-null mouse embryonic fibroblasts treated with HU. Also, we showed that siRNA-mediated silencing of cyclin D1 or Cdk4 suppressed H-Ras-^G12V ^or H-Ras^G12V^/c-Myc-dependent centrosome amplification in MCF10A human mammary epithelial cells, while inhibition of cyclin E or cyclin B did not prevent centrosome amplification [22].

An important molecule downstream of Cdk2 that restricts centrosome separation and duplication is NPM phosphorylated at residue T199 [96,97,157]. Reasoning that this mode of deregulation was an important intermediate to centrosome amplification, our group showed that when E2F3a/b is ablated, cyclin E/Cdk2 activity is elevated, leading to the hyperphosphorylation of NPM^T199 ^[33]. Hyperphosphorylation of NPM^T199 ^by Cdk2 strongly correlated with constitutive centrosome duplication cycle and centrosome amplification. The role of NPM as a negative regulator of centrosome duplication was confirmed genetically through a gene knockout approach, as cells heterozygous for NPM displayed centrosome amplification [95]. Silencing of NPM in p53^-/-^p19Arf^-/-^Mdm2^-/- ^MEFs also resulted in centrosome amplification [158]. In the same system, ectopic expression of NPM^T198A ^could not rescue the centrosome amplification phenotype in p53^-/-^p19Arf^-/-^Mdm2^-/- ^MEFs. In contrast, our group used a similar mutant of NPM, NPM^T199A ^(which cannot be phosphorylated by Cdk2 or Cdk4) to demonstrate that this mutant prevented centrosome amplification in p53-null cells to the same extent as ablated Cdk2 or Cdk4 [86]. These experiments demonstrated that the G_1 _phase Cdks signal centrosome amplification in p53-null cells through NPM. In terms of other mechanisms linking the G_1 _phase Cdks and centrosome amplification, the Fry group demonstrated that nuclear export is required for centriolar satellite formation and centrosome overduplication in p53-null cells, with export inhibitors causing a Cdk2-dependent accumulation of nuclear centrin granules [153]. This group proposed an interesting model of regulation of centriole reduplication: Centrosome precursors arise in the nucleus, providing a novel mechanistic explanation for how nuclear Cdk2 can promote centrosome overduplication in the cytoplasm.

Other than the hyperphosphorylation and inactivation of NPM and the nuclear accumulation of centrin intermediates, processes that are dependent on Cdk2, the centrosomal targets controlled by oncogenes and altered tumor suppressors directly responsible for centrosome amplification are largely unknown. The sole exception is Nek2; it has been observed that silencing Nek2 abrogated centrosome amplification in human mammary epithelial cells expressing H-Ras^G12D ^and H-Ras^G12D^/c-Myc [22]. Speculatively, we can propose the following model: Oncogene-activated G_1 _phase Cdks signal centrosome amplification through the stabilization of centrosome duplication kinases such as Plk4 or Mps1, or through E2F-dependent transcriptional deregulation of those centriole duplication kinases (Figure 1).

## Conclusions and future directions

Because centrosome amplification is present in the vast majority of human tumors, and since supernumerary centrosomes may generate aneuploidy and genomic instability suggests that centrosome dysfunction is a potentially important contributor to cancer biogenesis. However, we are far from demonstrating a causal relationship between centrosome amplification and mammalian tumorigenesis. The observations that various pre-malignant lesions harbor centrosome amplification first mapped centrosome amplification to tumor initiation. Recent evidence demonstrating that low level aneuploidy caused by interference with spindle assembly components causes various tumors in mouse models [159,160], together with observations that merotelic attachments cause that same kind of aneuploidy [161,162] helped to bridge the gap between the correlation of centrosome amplification, aneuploidy and tumor initiation. Furthermore, two recent manuscripts showed that ectopic expression of centrosome regulatory proteins leads to benign tumors in transplanted Drosophila brain stem cells, suggesting for the first time a direct relationship between centrosome amplification and tumorigenesis [23,24]. However, unlike mammalian cancers, which are grossly aneuploid, the benign tumors in Drosophila harboring centrosome amplification displayed neither aneuploidy nor detectable gross chromosomal aberrations [24]. The classic Weinberg experiments may help shed some light on the kind of genomic changes that may be needed to transform a human epithelial cell. For example, they showed that transformation of a primary human mammary epithelial cell required ectopic expression of telomerase to protect from senescence induced by telomere shortening [163]. Ectopic expression of Ras and c-Myc as well as inactivation of p53 and Rb (via the SV40 large T antigen) was also required for transformation, suggesting that some cooperation is necessary to transform primary cells. It is to note that most of the genes that were required to transform those mammary epithelial cells affect centrosome amplification, or allow the generation of chromosome breaks and recombination [22,134,135,155,164-168]. This suggests that the centrosome amplification and genomic instability triggered by those oncogenes, combined with their ability to affect proliferation provide those cells selective advantages to initiate mammary tumors. Future experiments are needed to understand how centrosome amplification transforms cells, and whether it eventually causes ectopic proliferation and decreases apoptosis, or whether it contributes to tumorigenesis by altering other processes, such as the orientation of cells within a tissue, a concept postulated by the Gonzalez group in their Drosophila model [24]. Another pressing issue is to establish, using proteomics and transcriptomics, the centrosomal targets that are deregulated by various oncogenic and altered tumor suppressive pathways. This will allow for the ectopic expression or inactivation of various centrosome regulatory proteins in primary cell lines to more directly assess the role of centrosome amplification in transformation.

## Competing interests

The authors declare that they have no competing interests.

## Authors' contributions

MKH participated in the design, research, writing and editing of this review. AA participated in the research and writing of this review. HS conceived the review and participated in the design, research, writing, and editing of this review. All authors read and approved the final manuscript.
